# Actionable Strategies to Target Multiple Myeloma Plasma Cell Resistance/Resilience to Stress: Insights From “Omics” Research

**DOI:** 10.3389/fonc.2020.00802

**Published:** 2020-05-15

**Authors:** Sabrina Manni, Anna Fregnani, Gregorio Barilà, Renato Zambello, Gianpietro Semenzato, Francesco Piazza

**Affiliations:** ^1^Department of Medicine, Hematology and Clinical Immunology Branch, University of Padova, Padova, Italy; ^2^Foundation for Advanced Biomedical Research – Veneto Institute of Molecular Medicine (FABR-VIMM), Padova, Italy; ^3^Department of Surgery, Oncology and Gastroenterology (DISCOG), University of Padova, Padova, Italy

**Keywords:** proteotoxic stress response, autophagy, replication stress, therapeutic targets, Omics analyses

## Abstract

While the modern therapeutic armamentarium to treat multiple myeloma (MM) patients allows a longer control of the disease, this second-most-frequent hematologic cancer is still uncurable in the vast majority of cases. Since MM plasma cells are subjected to various types of chronic cellular stress and the integrity of specific stress-coping pathways is essential to ensure MM cell survival, not surprisingly the most efficacious anti-MM therapy are those that make use of proteasome inhibitors and/or immunomodulatory drugs, which target the biochemical mechanisms of stress management. Based on this notion, the recently realized discoveries on MM pathobiology through high-throughput techniques (genomic, transcriptomic, and other “omics”), in order for them to be clinically useful, should be elaborated to identify novel vulnerabilities in this disease. This groundwork of information will likely allow the design of novel therapies against targetable molecules/pathways, in an unprecedented opportunity to change the management of MM according to the principle of “precision medicine.” In this review, we will discuss some examples of therapeutically actionable molecules and pathways related to the regulation of cellular fitness and stress resistance in MM.

## Introduction

Multiple myeloma (MM) is the most frequent neoplastic disorder affecting post-germinal center B cells and plasma cells, the final stage of B-lymphocyte differentiation (1–3). Despite the clinical severity and dismal prognosis that still characterize MM, the overall survival of affected patients has consistently improved over the last two decades (the 5-year survival rate has nearly doubled) thanks to the application of autologous stem cell transplantation, the use of novel agents and the introduction of maintenance therapy (2). New drugs that have substantially revolutionized the anti-MM therapies are proteasome inhibitors, immunomodulatory agents, and the anti CD38 and anti-SLAMF7 monoclonal antibodies directed against specific plasma cell surface molecules. More recently, a great deal of research efforts are being devoted to the immunotherapy with anti-B-cell maturation antigen (BCMA) Chimeric Antigen Receptor (CAR)-T cells or bispecific T cell engagers (2, 3). Nonetheless, MM remains a difficult-to-eradicate tumor because it displays a great predisposition toward biological heterogeneity and clonal evolution in time and space that ultimately confers resilience to stress and resistance to cytotoxic agents (4–8). Being the pathobiological features of MM as such, the identification of targets that sustain MM cell “invulnerability” seems a central research goal to pursue.

In this review, we have examined some facets emerging from the body of high-throughput data of functional genomics, transcriptomics, gene silencing, and drug screen that deal with potential vulnerable targets of MM biology liable to therapeutic targeting. We will first discuss the pathways active in MM involved in the management of the proteotoxic/autophagic stress and the replicative/oxidative stress and then analyze the available data coming from -OMICS and functional screens that may allow to design novel therapeutic approaches targeted against stress-managing mechanisms.

## Chronic Stress and Pathways of Stress Management in MM

### Endoplasmic Reticulum (ER) Stress/Unfolded Protein Response (UPR) and Autophagy

**Figure 1:**
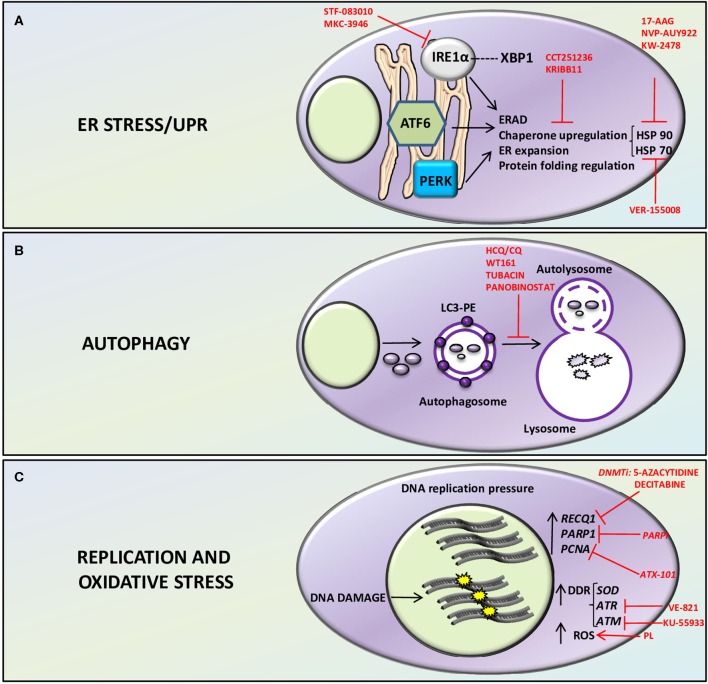
Therapeutically relevant Stress related pathways in MM. ER stress/UPR **(A)**, autophagy **(B)**, and replication and oxidative stress **(C)** related pathways and putative therapeutic targets in MM plasma cells. **(A)** the ER, resident stress sensors IRE1α, PERK, and ATF6 are activated by accumulation of misfolded proteins and sustain MM cell survival by ERAD (ER associated protein degradation) and by protein folding regulation through upregulation of chaperones such as HSP70 and HSP90. Inhibition of HSP90 with 17-AAG, NVP-AUY922, KW-2478, or HSP70 with VER-155008 or both chaperones via inhibition of their regulator HSF-1 with the compounds CCT251236 or KRIBB11 has cytotoxic effect on MM cells. IRE1α inhibition with STF-083010 or MKC-3946 displayed anti-myeloma activity. **(B)** Autophagy, by recycling proteins and organelles avoids cell death. In particular, it conveys cellular components to lysosomes, through the formation of autophagosomes and autolysosomes with the activation of a series of autophagy related proteins (ATGs) and the conjunction of LC3-I with phosphatidylethanolamine (PE) to form the lipidated form LC3-II. Inhibitors of autophagy such as Hydroxychloroquine (HCQ)/Chloroquine (CQ), or HDAC inhibitors such as WT161, tubacin, and panobinostat has been employed to interfere with autophagy and hamper protein homeostasis, leading to MM cell death. **(C)** MM plasma cells display high DNA replication pressure with the consequent likelihood of exposition to DNA damage. MM plasma cells rely on high expression of replication related proteins such as PCNA, PARP, and RECQ1 helicase. DNA damage response (DDR) and oxidative stress are chronically activated in MM through the stimulation of DNA stress managing proteins such as ATR, ATM, SOD, and ROS production. RECQ1, PARP1, PCNA, and DDR inhibitors and compounds that interfere with oxidative stress are depicted in the figure. DNMTi, DNA methyltransferase inhibitors; PARPi, PARP inhibitors; PL, Piperlongumine.

The protein overload to which MM cells are subjected (due to their activity as antibody producing/secreting cells), is cause of a massive chronic proteotoxic stress, which needs to be managed (9–11). The ER is a chief organelle of accumulation of aberrant proteins and three main homeostatic stress-managing pathways are activated to avoid the potential damage from these misfolded proteins. The UPR-related, ER-resident stress sensors IRE1α, PERK, and ATF6 are activated by the accumulation of misfolded proteins (12, 13). Each of the sensors triggers a signaling cascade that leads to changes in the expression levels of chaperones and other enzymes in order to assist protein maturation or degradation (14) (Figure 1A). Moreover, autophagy, which is also essential for normal and malignant plasma cell development, may compensate in part for an impaired UPR/proteasome response by assisting the resolution of proteotoxic stress through the recycle of proteins and organelles and avoiding cell death (15, 16). In particular, autophagy conveys cellular components to lysosomes, through the formation of autophagosomes and autolysosomes with the activation of a series of autophagy related proteins (ATGs) such as ATG7, ATG8, ATG12, ATG5, ATG10, and the conjunction of LC3-I with phosphatidylethanolamine (PE) to form the lipidated form LC3-II (17) (Figure 1B). A functional node is represented by the proteasome, the cellular machinery in charge of the proteolysis of polypeptides, which warrants a correct protein turnover (18). However, an overwhelming or prolonged proteostatic/proteotoxic stress represents and Achilles's Heel that may eventually elicit apoptosis (14).

### Replication and Oxidative Stress

Recent evidence has highlighted the importance of the replication and oxidative stress in MM (19–21). Targeting the addiction to proliferating cell nuclear antigen (PCNA), which is overexpressed in MM and is essential for replication and DNA damage response, caused MM cell apoptosis, and growth arrest, while no effects were observed in non-malignant cells (19). It was also demonstrated that, due to the strong replicative pressure to which MM cells are subjected, a chronic activation of the DNA damage response occurs in MM cells (20). In these processes, the oncogene MYC plays a central role. Again, the addiction that malignant plasma cells develop to the replication and oxidative stress-managing pathways, i.e., to the ATR and SOD enzymes, accounts for the strong anti-proliferative and pro-apoptotic effect obtained by the inhibition of these two proteins (20). Similarly, MM cells are addicted to RECQ1 helicase, an enzyme involved in DNA unwinding and maintenance of chromosome integrity. RECQ1 is overexpressed in MM and its relationship with an enhanced resistance to replicative stress, could confer in turn a higher resilience of malignant plasma cells toward the cytotoxic effects of chemotherapy (22). Therefore, targeting this protein has been suggested as a potential strategy to increase MM cells susceptibility to replication stress and apoptosis (21).

Oxidative stress, a hallmark of cancer, has also been demonstrated to play a major role in MM. Malignant plasma cell intrinsic generation of reactive oxygen species (ROS) may emanate from genotoxic stress, replicative stress, and proteotoxic stress (5). Several studies that have investigated the expression of various regulators have demonstrated a status of oxidative stress in MM cells and blood samples from patients (23–26). It has also been shown that oxidative stress is modified upon conventional and novel anti-myeloma agents (27, 28). Moreover, expression/modulation of oxidative stress pathway components may influence the responsiveness of MM cells to certain cytotoxic agents (29–31). Recently, it was demonstrated that the cytotoxicity on MM cells of melphalan, a central chemotherapeutic in MM treatment, is partly mediated by the generation of oxidative stress and can be antagonized by antioxidant mechanisms. Glutathione, a physiological anti-oxidant agent, could reduce melphalan-induced apoptosis and cell cycle alterations but this effect was independent from melphalan-induced DNA damage (32).

## Genetics and Genomics of Multiple Myeloma and Pathways of Stress Management

Insights into the genomic landscape of MM came from a number of important studies that have investigated the disease's genome through whole exome or whole genome sequencing. In the paper by Chapman et al. (33), a first global analysis of malignant plasma cells from 38 newly diagnosed MM (NDMM) patients has been conducted. This report highlighted the most frequent mutations in NDMM by analysis of whole genome sequencing (WGS) of 23 cases and of whole exome sequencing (WES) of 16 cases (being one patient sample subjected to both experimental techniques). Confirming previous reports, *KRAS* and *NRAS* were the most frequently mutated genes found (overall in 50% of cases), followed by *TP53* (8%). To note, the clonal drift from *KRAS* to *NRAS* may confer a worse prognosis (34). Newly described mutations to *CCND1* (CyclinD1) gene were also detected in 5% of cases. One extremely interesting finding of this work was the frequent incidence of mutations affecting genes involved in cellular processes deeply connected with cellular stress management (such as RNA processing, protein translation, and the unfolded protein response) in roughly 50% of patients. The most frequently mutated genes were *DIS3/RRP44* in 11%, *FAM46C* in 13%, *LRRK2* in 8% of cases. Also, mutations in the gene coding for the transcription factor XBP1 (described above as instrumental for plasma cell development and function and ER stress response), have been recognized in 5% of cases. Altogether, 42% of cases were found to have mutations affecting protein homeostasis. Another interesting result was the finding of an accumulation of mutations to members of pathways involved in chromatin regulation, NF-κB transcription factor, and coagulation cascades. Eleven NF-κB pathway genes were found mutated: *BTRC, CARD11, CYLD, IKBIP, IKBKB, MAP3K1, MAP3K14, RIPK4, TLR4, TNFRSF1A*, and *TRAF3*. Also, the discovery of an impaired H3K27Me3 in the *HOXA9* gene with consequent aberrant upregulation of the expression of this transcription factor could be ascribed to mutations affecting histone methylation regulators *MLL, MLL2, MLL3, UTX, WHSC1, WHSC1L1*. These mutations are of pathogenetic importance in a subset of MM cases, since it was demonstrated that *HOXA9* overexpression may confer a growth advantage to MM cells (35).

Clinically actionable mutations to *BRAF* were also discovered in this first report, which analyzed many MM patient samples. The *BRAF* G469A mutation in one of the 38 patients and the *BRAF* K601N and *BRAF* V600E mutations in 4% of additionally sequenced 161 cases, point to a pathogenetic role of the BRAF regulated signaling, which could be targeted by BRAF inhibitors (36).

Subsequently, a refined analysis including copy number alterations that was powered (<30x sequence coverage) for detecting clonal heterogeneity, has been performed. A larger set (*n* = 203) of NDMM and treated MM patients' samples was examined, 177 by WES, and 26 by WGS (37). Eleven recurrently mutated genes were identified, some of which known (*NRAS, KRAS, TP53, FAM46C, DIS3*). Other genes were confirmed mutated (*BRAF, PRDM1, RB1, TRAF3, CYLD1*), which are known/believed genes to be of pathogenic importance in MM. The data from the earlier study relative to the pathway-level mutations were confirmed with regard to the NF-κB, coagulation cascades, and histone methylation pathways (37). Analysis of clonal (likely earlier) versus subclonal (later) mutations revealed that driver mutations are not always clonal. For instance, KRAS mutations were detected in 73% of cases as clonal and 27% of cases as subclonal events. Rarely, *KRAS, NRAS*, and *BRAF* mutations were found both in the same clone, while this was true for the *DIS3* and *KRAS* mutations. This finding has implications for targeted therapy. Indeed, analyzing *BRAF* mutations as actionable targets, it was demonstrated that while *BRAF*-mutated MM cells are sensitive to BRAF inhibition, a paradoxical growth-promoting effect of BRAF inhibitors is present in MM cells with WT *BRAF*, due to a hyper-activation of the MEK/MAPK pathway (37).

Another approach was used in a mix confirmation/discovery study in NDMM, in which it was confirmed that in MM there are commonly mutated genes and a long tail of uncommonly mutated genes (38). The NF-κB pathway and the DNA damage (*TP53, ATM, ATR, BRCA2*) response pathways were confirmed and identified as recurrently mutated. To note, when the data were interrogated in the context of proteasome inhibitor treatment, no correlations could be found with any alterations, perhaps due to the relatively small sample size (38).

Walker et al., through the use of integrated genomics investigated the mutational landscape, copy number variations, primary translocations and hyperdiploid status in a large cohort of 1,273 newly diagnosed MM patients (derived from the Myeloma XI trial, the Dana-Faber Cancer Institute/Intergroupe Francophone du Myelome, and the Multiple Myeloma Research Foundation CoMMpass study), finding 63 MM driver genes. Among them, some were already previously known (such as *FGFR3, DIS3, FAM46C, MAF, BRAF, MYC, CCND1, ATM, IRF4, PRKD2, NF-KB* signaling pathways related genes), some others were new, (such as *IDH1, IDH2, HUWE1, PTPN11, KLHL6)* (39).

Bolli et al. (40) have also characterized the genomic landscape of 11 smoldering MM (SMM) by WGS. This analysis has detected on average of 5,308 mutations and 4,397 small *indels* per patient. Important findings included the frequent MYC translocation with non-immunoglobulin heavy (IgH) chain locus partner (5/11) and the overall pattern of driver alterations similar to overt MM, indicating a clear earliness of their onset during myelomagenesis (40). Analysis of a significant interaction between driver events revealed two associations, between *PRDM1* deletions and *t*(4, 14), which confers a worse OS and between *PRDM1* deletions and *BIRC2/3* deletions, which confers a better OS.

Maura et al., through WGS data of 67 MM genomes from 30 patients collected at different times, in association with whole exome data from 804 patients within the CoMMpass trial (NCT01454297) deeply delineated MM genomic subgroups, taking into consideration the mutational landscape, copy number variation, and structural variants. The authors identified 55 distinct genes altered, and among others, they revealed novel driver mutations in *ABCF, ZFP36L1, TET2, ARID2, KDM6A*, and *EP300* genes and in the linker histones HIST1H1B, HIST1H1D, HIST1H1E, and HIST1H2BK. They next chronologically reconstructed in a comprehensive manner, driver events in MM pathogenesis (41).

Vikova et al. (42) analyzed through WES, the molecular signature of 30 MM cell lines and 59 primary MM tumors, comparing with eight control samples revealing different mutated driver genes and pathways associated to drug resistance. Novel mutated genes were linked to mitosis, DNA repair processes, chromatin remodeling, and epigenetic modifiers, (such as *CNOT3, KMT2D, SETD2, MSH3, PMS1, EZH2*), protein trafficking (such as USP6) and altered signaling cascades were associated to the PI3K/AKT (mutations in *TSC1, TSC2, TBX3, PTEN, IKBKB* genes), MAP kinase (*MAP2K2, RAC1, RAF1, NF1* mutated genes), JAK/STAT (*STAT3, RUNX1, EPAS1, JAK2, STAT6* mutated genes) P53/cell cycle (*TP53, ATM, CCND1, RB1, CDKN2A*) some of which are potential targets from the therapeutic point of view. *KMT2D and SETD2* were mutated only in patients at relapse. Moreover, some mutated genes were associated to drug resistance, such as *FAM46C* and *KRAS* (panobinostat), *KMT2D* (dexamethasone), *PMS1* (TSA), and USP6 (SAHA). *KMD2* mutations were also related to lenalidomide sensitivity.

Tessoulin et al. (43) through WES of human MM cell lines found driver genes related to chromatin regulation/modification and DNA repair, associated to drug resistance.

Altogether, these OMICS data suggest that targeting stress associated pathways such as DNA damage response or epigenetic modifiers could offer therapeutic alternatives in MM.

## Transcriptomics of MM and Pathways of Stress Management in MM

Transcriptomic analysis has been applied to MGUS, SMM, and MM and has identified associated gene expression signatures (44). Gene expression studies have also led to the recognition of the cyclin D overexpression signature as a common feature of MM (45) and of the chromosome 1 transcriptional deregulations as prognostic alterations in high risk MM (46).

Molecules mis-expressed in MM belong to different pathways and functions, including stress-managing pathways, UPR/ER stress, proteasome, and mitochondria function. Recently, the work of Jang et al. (47) was able to dissect the spatial and temporal heterogeneity of MM plasma cellular clones using scRNA-Seq expression profiling at the single cell level in 15 plasma cell dyscrasia patients that included 3 MGUS, 4 SMM, 5 NDMM, and 3 relapsed/refractory MM (RRMM) analyzing a total of 597 cells. The authors identified subpopulations of cells within the same patient sample that express different levels of the same gene, accounting for the heterogeneity expression profiling of different plasma cells within a patient. Cells were clustered into four subpopulations (L1–L4) according to gene expression, being cells in L1 group identified by the lowest expression of genes involved in oxidative stress, MYC target, and mTORC1 dependent signaling pathway. Interestingly, the expression profiles within the four groups correlated with disease progression, most of the cells belonging to MGUS patients clustering in the L1 subgroup. Indeed, this subgroup showed the lowest expression of genes linked to cell metabolism and protein homeostasis, such as all the 18 genes coding for the proteasome subunits, UPR related genes, and genes associated to mitochondria metabolism and function. The authors identified a signature of 44 genes that are consistently related to MM progression. Among these, 26/44 (59%) were linked to UPR/ER stress (such as *ARF1, ATF6, EIF2a, ERLEC1, CD46, BSCL2, CDK2AP2, IER3IP1, IFNAR1, PSMB1, SLAMF1, SSR2)* and mitochondria (such as *ATP5G1, ATP5J, DAP3, GNG5, JTB, ROMO1*), underlying the importance of stress managing genes expression in the disease progression (47). Of note, kinases such as *CSNK1A1* and *CSNK2B*, which were shown to be essential for MM plasma cell survival and proteotoxic stress handling (48–50), have been found altered within the four groups, with increasing expression from L1 toward L4. Also, autophagic gene expression (such as *ATG3*) has been found altered among the groups. ATG3, is important for LC3 lipidation, and therefore is essential for autophagocytosis.

Heat shock proteins are essential chaperones that ensure the correct protein homeostasis and folding and helped the management of MM stress, due to hyperactivity of the protein machinery in the antibodies secreting malignant plasma cells. It has been shown that chaperone genes such as *HSPA9* and *HSPE1* coding respectively for GRP95/HSP70 and HSP10 are significantly differentially (higher) expressed in L2–L3–L4 groups compared to L1. Moreover, increased expression of protein homeostasis related genes in plasma cells in the L2–L3–L4 groups was linked to disease progression and reduced OS of MM patients.

Liu et al. (51) analyzed the gene expression profile, copy number variation, and clinical features in a large data set from the Multiple Myeloma Research Consortium (MMRC) identifying eight prognostic signatures encompassing 178 genes related to cell cycle progression and a molecular gene signature involved in immunomodulatory drugs and proteasome inhibitors response. The authors were able to create a MM molecular causal network model, by integrating gene expression and copy number variation data, with supposed key regulators, such as genes involved in cell cycle and metabolic pathways. The results not only identified genes already known to be altered in MM, such as translocations occurring between the heavy chain of immunoglobulins and known oncogenes (*CCND1, CCND3, MAF, FGFR3, MMSET*), but also two novel nodes composed by *Alkylglycerone Phosphate Synthase (AGPS*) and *Alpha Thalassemia/Mental Retardation Syndrome, X-Linked* (*ATRX*), which regulate multiple genes (41 and 32, respectively). The *AGPS* gene is involved in lipid biosynthesis, a process that many have shown to play a fundamental role in MM progression. Targeting AGPS could therefore be of potential benefit to increase MM cell death, since multiple AGPS inhibitors are under development in other cancers (52).

The *ATRX* gene has been involved in chromatin remodeling, and could be also therapeutically targetable in MM. It has been previously shown that it is a mutational driver (39). Moreover, altered genes were found in molecular pathways related to cell cycle, mitosis, macromolecule biosynthesis, DNA damage response such as *NOP16, CECR5, MELK* and *TPX2, NCAPG2, CDK1*, and *DTL*, for many of which there are already inhibitors available that could trigger MM cell apoptosis and cell cycle arrest.

Similarly, the authors identified a treatment response signature which is characterized by the deregulation of genes involved in protein folding and trafficking, such as *FKB5* and the HSP70 cochaperone *DNAJA1*, which could also be therapeutically relevant.

Altogether, once more, these results highlight the importance of the potential targeting of stress managing genes to increase plasma cell vulnerability, implementing MM therapy efficacy.

Table 1 shows a list of MM-related genes found altered in pathways essential for plasma cell dyscrasias through OMICS research.

**Table 1:** List of MM related pathways genes found altered in plasma cell dyscrasias through “OMICS” research.

**Altered MM pathways**	**Genomics or transcriptomics**
	
NF-κB signaling	BTRC, CARD11, IKBIP, IKBKB, MAP3K1, MAP3K14, RIPK4, TLR4, TNFRSF1A, (33) TRAF3, CYLD (33, 37, 39, 41, 43), TRAF2, NFKB1, NFKB2 (39), TNFAIP3, *CD74*, BIRC2, *BIRC3, IL2R4, NFE2L3* (43).
MAPK signaling	*BRAF* (36, 37, 39, 42), MAP2K2, RAC1, RAF1, NF1 (42).
PI3K signaling	TSC1, TSC2, TBX3, PTEN, IKBKB (42).
JAK/STAT signaling	STAT3, RUNX1, JAK3, STAT6, EPAS1 (42).
GTP ases	*KRAS, NRAS* (33, 37, 41–43).
ER stress/UPR/trafficking	*XBP1* (33), *ARF1, ATF6, EIF2a, ERLEC1, CD46, BSCL2, CDK2AP2, IER3IP1, IFNAR1, PSMB1, SLAMF1, SSR2, HSPA9, HSPE1* (47), *FKB5, DNAJA1* (51), *ABCF1* (41), USP6 (42).
Apoptosis/transcriptional regulators	*MAFB, MYC, MAX, HUWE1* (39), *MAF* (39, 41), BIRC2, *BIRC3, EGR1, LP1, BCL2L11, BIM* (43).
Autophagy	*LLRK2* (33), *ATG3* (47).
Replicative stress/DNA repair	*TP53* (33, 37–39, 42, 43) *CCND1* (33, 39, 41, 42) *H3K27Me3 in HOXA9* (33), RB1 (37, 42), ATM, ATR, BRCA2 (38, 40, 43), ATM, MSH3, PMS1, MSH3, CDKN2A (42), *FANCI, FANCA, FANCD2, RECQL4, RECQL5, BLM* (43).
RNA processing	*DIS3/RRP4, FAM46C* (33, 37, 39, 41–43), SF3B1 (39)
Cell cycle/mitosis/chromatin remodeling	*ATRX, NOP16, CECR5, MELK, TPX2, NCAPG2, CDK1, DTL* (51), *CDNK1B, FUBP1* (39, 41), ARID2, KDM6A, EP300, HIST1H1B, HIST1H1D, HIST1H1E, HIST1H2BK, *MMSET* (41), CDKN2C (41, 43) *CNOT3, KMT2D, MN1, EZH2* (42), *SETD2 PALB2, HDAC7, DOT1L* (42, 43), *TET2, PTPN11*, PRKD2 (39, 41, 43).
Oxidative stress	*ROMO1* (47), *BLVRB* (51).
Immune function	*KLHL6, IRF4, LTB, PRDM1* (39, 43).
Lipid metabolism	*AGPS* (51).

## Other OMICS: RNAI, CRISPR/CAS9, and Drug Functional Screening in MM

Other approaches have been employed in the search of new therapeutic targets in MM and to overcome drug resistance. Targeted transcriptome/genome editing (RNAi or CRISPR/CAS 9) or high- throughput cell-based drug screening have been developed to test novel druggable targets. Such screenings have also the potential to establish putative novel regulators of immunomodulatory drug or proteasome inhibitor sensitivity.

Zhu et al. (53) transfected a library of 27968 RNAi in MM cells to determine lenalidomide sensitizers, identifying 63 genes that empowered lenalidomide activity upon silencing.

Among others, Ribosomal protein S6 kinase (RPS6KA3 or RSK2), five RAB family members, three potassium channel proteins, two peroxisome family members, I-k-B kinase-a (CHUK), and the transcription factor CREB1 were found the most sensitizing. Specific functional validation of RSK2 inhibition with RNAi or chemical inhibition not only sensitized MM cells to lenalidomide, but also to bortezomib, melphalan, or dexamethasone, pointing to a promising molecular target in MM therapy. Liu et al. (54) through genome wide CRISPR/CAS9 screening, identified seven out of nine of the CSN9 signalosome complex subunits as regulators of immunomodulatory drugs (IMIDs) sensitivity, by modulating the lenalidomide target Cerebron (CRBN) expression. Specific functional knock out of each of these *CSN* genes lead to partial pomalidomide and lenalidomide resistance, determining CRBN protein reduction.

Another example of how high-throughput research can help the identification of stress related targets, comes from the work of Stessman et al. (55) in which it was performed high-throughput drug screening in bortezomib sensitive and resistant cells. Among 1,600 small molecule compounds, 12 molecules were identified as effective in all the tested groups, among which four were toxic on bortezomib resistant cells or were able to restore their bortezomib sensitivity. The further functional assays were performed on the compound NSC622608, which was demonstrated to cause MM cell death through the modulation of TP53 signature, with the upregulation of the P53 dependent P21, NOXA, and PUMA proteins, the upregulation of *MT1H, HMOX1*, and *ANXA2* genes and the reduction of *POLD2, MCM5, MCM4, MCM3, MCM2, KIAA0101*, and *CCNA2* genes.

A tentative approach to link high-throughput drug screening with gene expression profiling and mutational analysis, has been presented at the 2019 ASH meeting. Coffey et al. (56) tested simultaneously 170 compounds and their target inhibitors along with NGS profiling to predict sensitivity to drugs. The registered clinical trial NCT03389347 (https://clinicaltrials.gov/ct2/show/NCT03389347) will analyze the feasibility of using high-throughput drug sensitivity and genomics data to evolve personalized treatments.

RNAi, CRISPR/CAS9, and small molecule drug screening are therefore an emerging field for the discovery of MM vulnerable targets, but to date the results on stress related pathways are scarce and more experimentation is underway.

## Capitalizing on the Information from “-OMICS” Toward Actionable Targets

The body of data obtained from high-throughput transcriptomic and genomic analyses has allowed to better elucidate the major pathobiological MM alterations. However, for these research achievements to be clinically useful, it is important to identify molecules/pathways most suitable for therapeutic targeting. In this regard, the recurrently altered mechanisms involved in cellular stress resistance and resilience against certain pro-death stimuli, which have been described in MM, could represent a groundwork to design new therapies.

### Targeting MM-Associated Anomalies in Molecules Involved in the Management of Protein Synthesis, ER Stress, and Autophagy

Malignant plasma cells are addicted to both UPR and autophagy and the inhibition of the proteasome is now a well-established step in the therapy of MM (57, 58). It is now believed that targeting UPR and/or autophagy regulating proteins may further contribute to MM cell apoptosis (9, 59, 60).

IRE1α is a peculiar enzyme endowed with a kinase and endoribonuclease activity (61, 62). It has been demonstrated that the levels of XBP1 transcription factor, which regulates the IRE1α-dependent branch of UPR, are important to confer bortezomib resistance (63). Indeed, the IRE1α-XBP1 axis seems a suitable therapeutic target for this disease (64–66). The inhibition of IRE1α endoribonuclease domain and therefore of XBP1 splicing was also proposed as a promising strategy to reduce the MM cell capacity of coping with the proteotoxic stress and kill MM cells (65, 66). The small compound STF-083010 displayed antimyeloma activity *in vitro* and *in vivo* (65) and the molecule MKC-3946 was able to stop the bortezomib and HSP90 inhibitors-induced ER stress with consequent increased cell death due to decreased XBP1 splicing and increased GAD153 levels (66). The potential beneficial effects of interrupting the IRE1α/XBP1 axis in MM have also been described in other studies, in which this pathway was impaired by manipulating upstream molecules acting as regulators (48).

The PERK/GADD153/eIF2α branch of the UPR is believed to regulate survival or apoptosis depending on the magnitude of its activation. Earlier studies demonstrated that it was possible to enhance the GADD153/eIF2α-dependent pro-apoptotic arm of the UPR by stopping eIF2αdephosphorylation (67). Using *in vitro* and *in vivo* models, it was shown that this perturbation of UPR was associated to a progressive elimination of bortezomib-resistant/G0-G1 cell cycle-arrested MM cells (67). In another study, it was shown that the down-modulation of the PERK axis causes a non-apoptotic cell death triggered by autophagy (68). Other means of perturbing this pathway have targeted ER stress/UPR upstream regulative kinases, such as CK2, with the result of causing a strong activation of PERK-mediated phosphorylation of eIF2α and consequent irreversible pro-apoptotic UPR (48). Interestingly, it was also shown that blocking the PERK or ATF4-elicited UPR may cause tumor growth arrest and a reduction of neoangiogenesis after glucose deprivation (69).

Targeting the chaperone machinery has also been therapeutically relevant in MM. Preclinical studies (48, 70–72) or clinical trials have been conducted using HSP90 inhibitors such as 17-AAG, NVP-AUY922, KW-2478 alone, or in association with bortezomib or dexamethasone in MM (ClinicalTrials.gov Identifier: NCT00514371 and NCT00546780 for 17-AAG, ClinicalTrials.gov Identifier: NCT00708292 for AUY922 and ClinicalTrials.gov Identifier: NCT01063907 for KW-2478). Moreover, it has been shown that HSP70, a protein which OMICS data have demonstrated to be altered across the stages of MM progression (see above), is a chaperone of HSP90, that mediates drug resistance in MM sustaining plasma cell survival. The concomitant inhibition of both HSP70 (with VER-155008) and HSP90 (with NVP-AUY922) increases MM cell death abolishing HSP70 upregulation induced by HSP90 inhibition and affecting PI3K-dependent MM survival signaling (73). HSP70 inhibition induced plasma cell apoptosis accumulating proteotoxic stress (17), causing changes in polyubiquitination, in ER stress/UPR protein expression, and chaperone related autophagy markers (such as LAMP-2A) (74–77). Therefore, a lot of efforts have been made to develop inhibitors that could target both chaperones. To this aim, CCT251236 or KRIBB11, novel Heat Shock Factor 1 (HSF1) inhibitors, have shown cytotoxic effects in MM cells, via induction of UPR, with altered EIF2α phosphorylation, CHOP expression and a reduction in protein synthesis (78). Thus, chaperone targeting seems a promising approach for the treatment of MM.

Autophagy inhibitors are also currently employed in clinical trials in MM. Hydroxychloroquine (HCQ)/Chloroquine(CQ) have been tested to increase the effects of proteasome inhibitors (bortezomib) preclinically (79) or in clinical trials underway in association with cyclophosphamide and dexamethasone (ClinicalTrials.gov Identifier: NCT01438177). Histone Deacetylases (HDAC) are deacetylating proteins that catalyze the excision of acetyl groups on Lys in given proteins, not limited to histones, providing further levels of control in protein homeostasis. HDAC6, in particular, has been involved in the autophagic process, by promoting aggresome formation and autophagosome-lysosome fusion (80). It has therefore been proposed to use HDAC6 inhibitors to interfere with autophagy and hamper protein homeostasis in MM. HDAC6 inhibitors such as WT161 and tubacin have displayed anti-MM cytotoxicity, modulating ER stress/UPR signaling events, overcoming proteasome inhibitors resistance (81, 82). Different HDAC inhibitors, such as panobinostat, a pan HDAC inhibitor, have been tested in clinical trials or have been approved in relapsed/refractory MM in association with bortezomib (83). Other more recently described autophagy modulators are protein kinases. In particular, our and others' laboratory work has described the role of protein kinase CK1α and CK1δ in the autophagic process in MM (84). It has been shown that the CK1α and CK1δ members of the CK1 family of S/T kinases may control the autophagic flux downstream oncogenic RAS as well as its tonic rate, thus impacting on the survival capability of MM plasma cells (84–86).

### Targeting MM-Associated Anomalies in Molecules Involved in the Management of DNA Damage-Induced Stress

Recent work has highlighted the importance of replicative stress management for myeloma cell survival. Cottini et al. (20) described a subset of aggressive myeloma displaying DNA damage due to chronic replicative and oxidative stresses, in part caused by the high activity of c-MYC. Remarkably, the concomitant inhibition of the DNA damage induced repair kinase ATR along with the blockade of ROS-triggered stress managing enzyme SOD, exerted a synthetic lethality on this aggressive subtype of MM cells (20).

In the paper by Viziteu et al., it has been demonstrated a dependence of MM cells on RECQ1 helicase, a DNA unwinding enzyme essential for chromosomal integrity. This enzyme is overexpressed in MM cells and protects from melphalan and bortezomib-induced DNA damage and cytotoxicity. Interestingly, through a miRNA-203-dependent pathway RECQ1 expression is downregulated after treatment with DNA methyl transferase inhibitors (21), thus representing a potential target for combined treatments.

## Conclusions

The information generated by the different high-throughput research on molecules and pathways affected in MM pathogenesis and evolution, have allowed to depict a very complex and heterogeneous scenario. Within this picture, it has been possible to identify some common alterations in cellular molecules/processes/mechanisms, which belong to the stress-related homeostatic response. It is becoming increasingly clear that some of these processes may be efficiently targeted for a therapeutic purpose, especially in combination with other approaches (Figure 1). Future research should focus on these molecules and validate their targeting as effective to achieve a clinically meaningful anti-MM action.

## Author Contributions

FP and SM conceived and wrote the paper, collected data, and envisaged the format. AF, GB, RZ, and GS wrote parts of the paper, contributed data, and insights.

## Conflict of Interest

The authors declare that the research was conducted in the absence of any commercial or financial relationships that could be construed as a potential conflict of interest.
